# Development and Clinical Translation of a Perioperative Nomogram Incorporating Free Fatty Acids to Predict Poor Outcome of Aneurysmal Subarachnoid Hemorrhage Following Endovascular Treatment

**DOI:** 10.3389/fneur.2021.629997

**Published:** 2021-07-26

**Authors:** Yongyi Wang, Yongfan Xie, Houzhong Wang, Jifang Zhang, Chuanfeng Li, Feng Chen, Chengtao Ren, Zhiming Xu, Mingxing Liu, Luo Li, Tong Li, Weimin Wang

**Affiliations:** ^1^Department of Neurosurgery, Qingdao Municipal Hospital, School of Medicine, Qingdao University, Qingdao, China

**Keywords:** endovascular treatment, clinical outcome, nomogram, free fatty acid, aneurysmal subarachnoid hemorrhage

## Abstract

**Objective:** A reliable prediction of clinical outcome is important for clinicians to set appropriate medical strategies in treating patients with aneurysmal subarachnoid hemorrhage (aSAH). In this study, we aim to establish a perioperative nomogram involving serum lipid signatures for predicting poor outcomes at 3 months in patients with aSAH following endovascular therapy.

**Methods:** Data of patients with aSAH receiving endovascular therapy were collected. Univariable and multivariable analyses were performed to screen independent predictors related to unfavorable outcomes defined by the modified Rankin Scale (mFS) ≥3. A novel nomogram based on these significant features was conducted. The clinical application of this nomogram was assessed by decision curve analysis (DCA) and clinical impact curve.

**Results:** A total number of patients included in this study were 213 (average age 58.9 years, 65.7% female), representing a poor 3-month outcome rate of 48.8%. Free fatty acid (FFA) levels on admission were efficient in predicting poor outcomes compared with other contents in serum lipids. Univariable and multivariable analyses revealed advanced age (*P* = 0.034), poor Hunt Hess (HH) (odds ratio, OR = 3.7, *P* < 0.001) and mFS (OR = 6.0, *P* < 0.001), aneurysms in the posterior circulation (OR = 4.4, *P* = 0.019), and higher FFA levels on admission (OR = 3.1, *P* = 0.021) were negative independent predictors of poor 3 months outcome. A novel nomogram composed of these significant features presented a concordance index (C-index) of 0.831 while the practical benefit was validated by DCA and clinical impact curve. An online calculator based on R programming promoted the clinical application of this nomogram.

**Conclusion:** Nomogram involving age, HH grade, mFS, aneurysm location, and serum FFA levels was sufficient to provide an individualized prediction of 3-month poor outcome for each patient with aSAH who underwent endovascular therapy.

## Introduction

Aneurysmal subarachnoid hemorrhage (aSAH) is a devastating stroke with high mortality and morbidity rate (1). As two large clinical trials showed that endovascular coiling could lead to longer disability-free survival and fewer worse outcome compared with neurosurgical clipping in treating ruptured intracranial aneurysms (2, 3), endovascular management of aSAH has been the first-line therapy in many clinical centers (4); however, despite its advantage over surgical clipping, endovascular coiling therapy still has an inherent risk of poor clinical outcomes due to the sudden onset of severe complications (5, 6). To reduce complications and improve aSAH recovery, clinical variables, such as premorbid history, radiological examination, and laboratory tests, collected at the first admission to medical centers were widely reported to be able to predict outcome following endovascular treatment, guiding the neuro interventionalists to provide essential medical supports to inpatients (7, 8). Unfortunately, the variables mentioned above are not enough to generate the accurate prediction of the poor clinical outcome, significantly limiting the clinical translation. Thus, it is critical to establish better predictive models to identify the patients at risk of poor outcomes based on more comprehensive clinical and economic factors, further guiding the appropriate inpatient management.

The roles of dyslipidemia in aSAH have been debated for decades, where the most of studies mainly focused on its contribution to the occurrence of aSAH. A meta-analysis on 21 previous literature revealed that the male patients with elevated total cholesterol (TC) levels would have a higher risk of SAH (9). The largest case-control clinical study to date also reported that both a higher high-density lipoproteins cholesterol (HDL-c) level and the administration of lipid-lowering agents are associated with a significantly lower risk of incidence of aSAH (10). The HDL-c level could contribute to predicting the growth and rupture of intracranial aneurysms (11). Inflammation was considered as a crucial pathophysiological mechanism leading to the poor outcome in SAH (12), while abnormal lipid profile facilitates the capacity of inducing the persisted neuroinflammation (13). Unfortunately, few studies have focused on the role of abnormal lipid profiles in predicting the clinical outcome of aSAH following endovascular treatment. Validated models including dyslipidemia parameters suitable for aSAH outcome prediction are relatively scarce.

In the current study, we conducted a retrospective analysis of the predictive values of serum lipid profile in patients with aSAH treated with endovascular coiling. In addition, we established a nomogram model incorporating significant risk factors of dyslipidemia for specialized use in predicting 3-month poor outcomes of these patients.

## Methods

### Study Population

Medical records of patients diagnosed with SAH in our stroke emergency center from September 1, 2015, to December 1, 2019, were retrospectively reviewed. The following were the inclusion criteria: (1) admitted to an emergency room within 24 h of first symptom onset; (2) aSAH confirmed by computed tomography (CT) and digital subtraction angiography (DSA); (3) received endovascular coiling for ruptured aneurysm within 24 h; (4) blood samples including lipid profile extracted before surgery; (5) age ≥18. The following were the exclusion criteria: (1) angiographically negative SAH; (2) treated by surgical clipping or declined to surgical intervention; (3) SAH that was not attributed to the ruptures of intracranial aneurysms (e.g., traumatic SAH, arteriovenous malformation); (4) had prior systemic diseases including chronic or acute renal failure, liver cirrhosis, malignancy, and infections; (5) had rebleeding or died before surgery; (6) age <18 or pregnancy; (7) transferred to other clinical centers.

### Clinical and Laboratory Assessments

Demographic characteristics (age and gender) and vascular risk factors (smoking status, hypertension, and diabetes) were collected. Aneurysm features (size and location) were described according to the DSA records. HH grade and modified Fisher scale (mFS) were recorded as the evaluation tool for the severity of aSAH on admission.

Serum lipid profiles were routinely measured on admission. This laboratory spectrum is composed of TC, triglyceride (TG), HDL-c, low-density lipoprotein cholesterol (LDL-c), and free fatty acids (FFA). As TG to HDL-c ratio (TG/HDL-c) (14) and non-high-density lipoprotein cholesterol (non-HDL-c) (15) were reported to be valuable parameters to predict the outcome among patients with stroke, we also took these relative indicators into the analysis.

We hypothesized that abnormal lipid contents triggered inflammation, therefore, neutrophil count, lymphocyte count, and monocyte count were investigated by automatic blood cell counters to calculate the neutrophil-to-lymphocyte ratio (NLR), monocyte-to-lymphocyte ratio (MLR), and systemic inflammation response index (SIRI = neutrophil count ^*^ monocyte count/lymphocyte count) because these parameters have been confirmed as novel biomarkers of systematic inflammation after aSAH and associated with complications and functional outcome in patients with aSAH (16, 17).

### Outcome Data

All participants received endovascular coiling and were confirmed to receive standard management in accordance with guidelines after endovascular interventional surgery (18). The primary parameter for measuring functional outcome is the modified Rankin score (mRS). The mRS ≥3 or death was referred to poor outcome (19). The outcome was recorded at 3 months after the occurrence of aSAH by two different neuro interventionalists in a regular out-patient or phone call follow-up.

### Missing data

During data collection, three patients died before DSA so the aneurysm parameters were missing. In addition, the mRS scores of seven patients were absent because of death (three patients) or failure to follow up (four patients). As the rate of missing data (4%) was statistically acceptable (20), patients with complete data were involved in analysis, while missing data were excluded.

### Statistical Analysis

All statistical analyses were performed on the commercial software SPSS version 26.0. Categorical variables were recorded as percentages. Continuous variables were presented as mean ± SD, and other continuous variables were expressed as median with the interquartile range and comparison. Differences in baseline data were analyzed using unpaired *t*-test or Mann-Whitney *U*-test for continuous variables, while the chi-square test or Fisher's exact test for categorical variables were appropriate. The variance inflation factor (VIF) and tolerance were used to check multicollinearity between clinical variables. Correlation between FFA and inflammation parameters (NLR, MLR, and SIRI) were determined by Pearson correlation coefficient analysis. Spearman rank-order correlation was used to test the correlation between significant lipid contents and HH grade. A univariate logistic regression tested the risk factors for an unfavorable outcome following endovascular treatment, variables with a *p*-value of <0.2 were entered into the multivariate logistic regression model to identify predictors of an unfavorable outcome. Odds ratio (OR) with 95% confidence interval (CI) were presented as results. Receiver operating characteristic (ROC) curves and areas under the curves (AUC) determined the predictive values of significant predictors. A nomogram based on independent predictors from the multivariate logistic regression model was established using R 4.0.1 for Windows. *DynNom* and *Shiny* packages were exploited to generate an online calculator, which can directly output predictive rates of unfavorable outcomes (https://www.shinyapps.io/). The performance of the nomogram was assessed by concordance index (C-index), and internal validation with 1,000 repetitions bootstrap resampling was used to test discriminative value. The calibration was confirmed by the Hosmer-Lemeshow test. Decision curve analysis (DCA) and clinical impact curve were conducted to evaluate the clinical utility of the nomogram. A 2-tailed *P* < 0.05 was considered statistically significant.

## Results

### Demographic Characteristics

**Figure 1:**
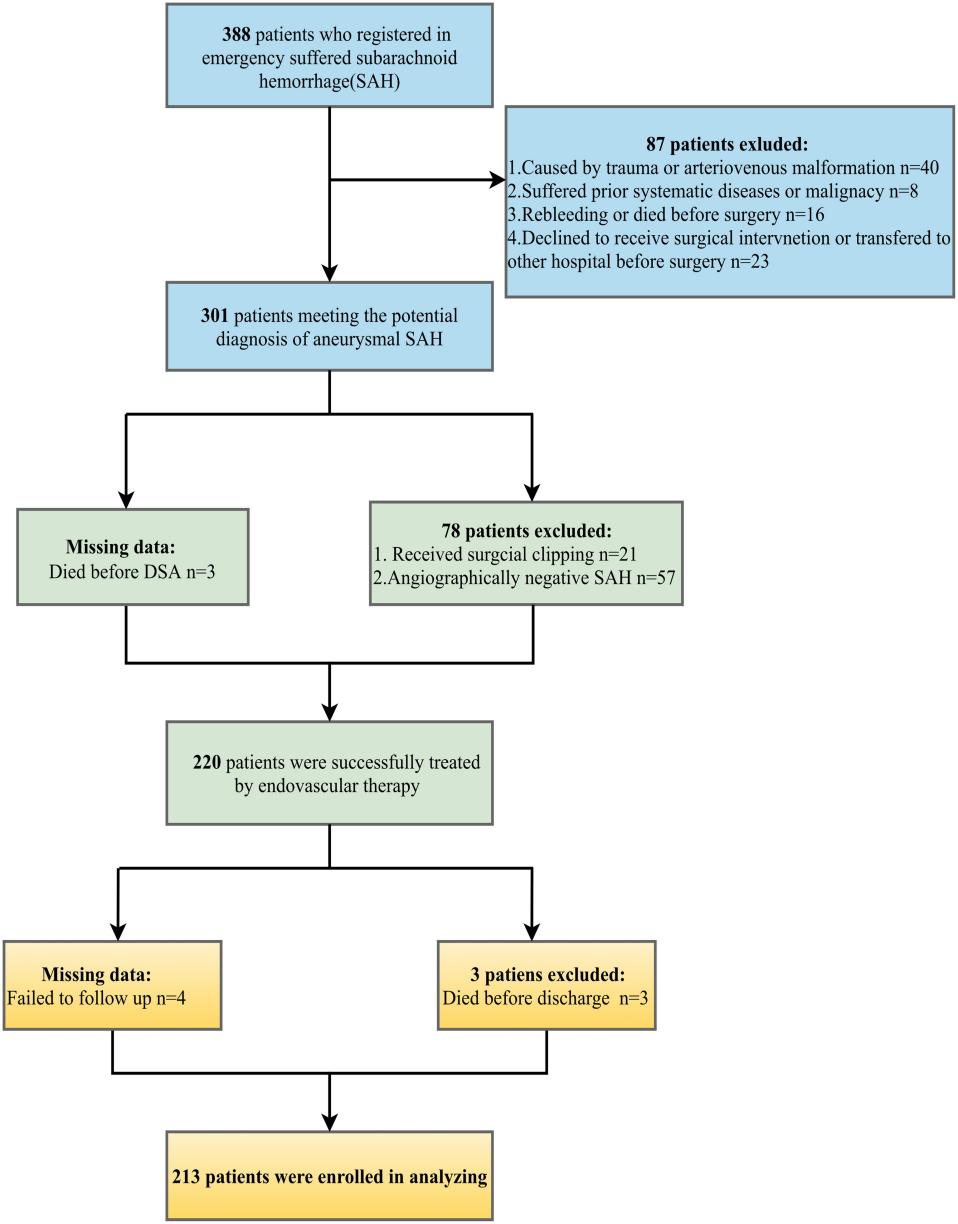
Flow chart outlining inclusion and exclusion criteria.

The demographic characteristics of patients are detailed in Table 1. We reviewed 388 patients with SAH records. Following the process of the inclusion and exclusion criteria in Figure 1, a total of 213 patients were eligible for this study, the majority of which were female patients (65.7%) with an average age of 58.9 years. Of these patients, 104 (48.8%) endured poor functional outcomes (mRS ≥3) at the 3-month follow-up. Premorbid history of smoking, hypertension, and diabetes mellitus were 22 (10.3%), 122 (57.3%), and 19 (8.9%) respectively. Assessments of the severity of initial aSAH showed 122 (57.3%) patients suffered high HH grade and 128 (60.1%) patients exhibited grade 3–4 of mFS by CT scanning. According to DSA records, most intracranial aneurysms were located in anterior circulation (190/213, 89.2%) compared with those in the posterior circulation (23/213, 10.8%). The diameter of the ruptured aneurysm was measured and classified into three groups, <5 mm (44.1%), 5–10 mm (49.8%), and ≥10 mm (6.1%).

**Table 1:** Comparison of demographic and clinical variables in patients with aSAH on admission and 3 months poor outcome treated by endovascular coiling.

**Parameters**		**3 months outcome**		***p-*value**
		**mRS <3**	**mRS ≥3**	
Number of patients	213 (100)	109 (51.2)	104 (48.8)	
Demographic characteristics				
Age^†^ (years)	58.9 ± 12.1	55.7 ± 11.5	62.2 ± 12.0	**<0.001**
Gender^‡^ (female)	140 (65.7)	72 (66.1)	68 (65.4)	0.918
Clinical data^‡^				
Hypertension	122 (57.3)	53 (48.6)	69 (66.3)	**0.009**
Diabetes mellitus	19 (8.9)	10 (9.2)	9 (8.7)	0.894
Smoking	22 (10.3)	14 (12.8)	8 (7.7)	0.217
HH grade				**<0.001**
Grade 1–2	91 (42.7)	68 (62.4)	23 (22.1)	
Grade 3–5	122 (57.3)	41 (37.6)	81 (77.9)	
Radiological findings				
mFS^‡^				**<0.001**
Grade 1–2	85 (39.9)	68 (62.4)	17 (16.3)	
Grade 3–4	128 (60.1)	41 (37.6)	87 (83.7)	
Aneurysmal location^‡^				**0.011**
Anterior circulation	190 (89.2)	103 (94.5)	87 (83.7)	
Posterior circulation	23 (10.8)	6 (5.5)	17 (16.3)	
Diameter of aneurysm (mm)^‡^			0.620
<5	94 (44.1)	48 (44.0)	46 (44.2)	
5–10	106 (49.8)	56 (51.4)	50 (48.1)	
≥10	13 (6.1)	5 (4.6)	8 (7.7)	
Lipid variables^§^				
TC (mmol/L)	4.94 (4.39, 5.75)	4.96 (4.45, 5.78)	4.94 (4.28, 5.73)	0.644
TG (mmol/L)	1.05 (0.79, 1.50)	1.05 (0.74, 1.51)	1.05 (0.82, 1.38)	0.694
HDL-C (mmol/L)	1.25 (1.07, 1.48)	1.25 (1.04, 1.48)	1.25 (1.07, 1.49)	0.586
LDL-C (mmol/L)	2.87 (2.47, 3.42)	2.88 (2.50, 3.41)	2.87 (2.37, 3.42)	0.560
Non-HDL-c (mmol/L)	3.65 (3.11, 4.40)	3.65 (3.22, 4.43)	3.72 (2.96, 4.36)	0.475
TG/HDL-c	0.90 (0.56, 1.26)	0.91 (0.52, 1.22)	0.85 (0.61, 1.29)	0.849
Non-HDL-c/HDL-c	2.99 (2.36, 3.66)	3.00 (2.48, 3.73)	2.89 (2.25, 3.61)	0.297
FFA (mmol/L)	0.56 (0.40, 0.77)	0.52 (0.38, 0.71)	0.62 (0.44, 0.84)	**0.012**

*FFA, free fatty acids; HDL-c, high-density lipoproteins cholesterol; HH, Hunt Hess; LDL-c, low-density lipoprotein cholesterol; mFS, modified Fisher scale; non-HDL-c, non-high-density lipoprotein cholesterol; TC, total cholesterol; TG, triglyceride*.

† *Mean ± SD*;

‡ *percentage (%)*;

§ *median (25th, 75th)*.

*The bold value indicates that the p-value of the parameter is less than 0.05*.

### Association of Admission Serum Lipids With aSAH

**Figure 2:**
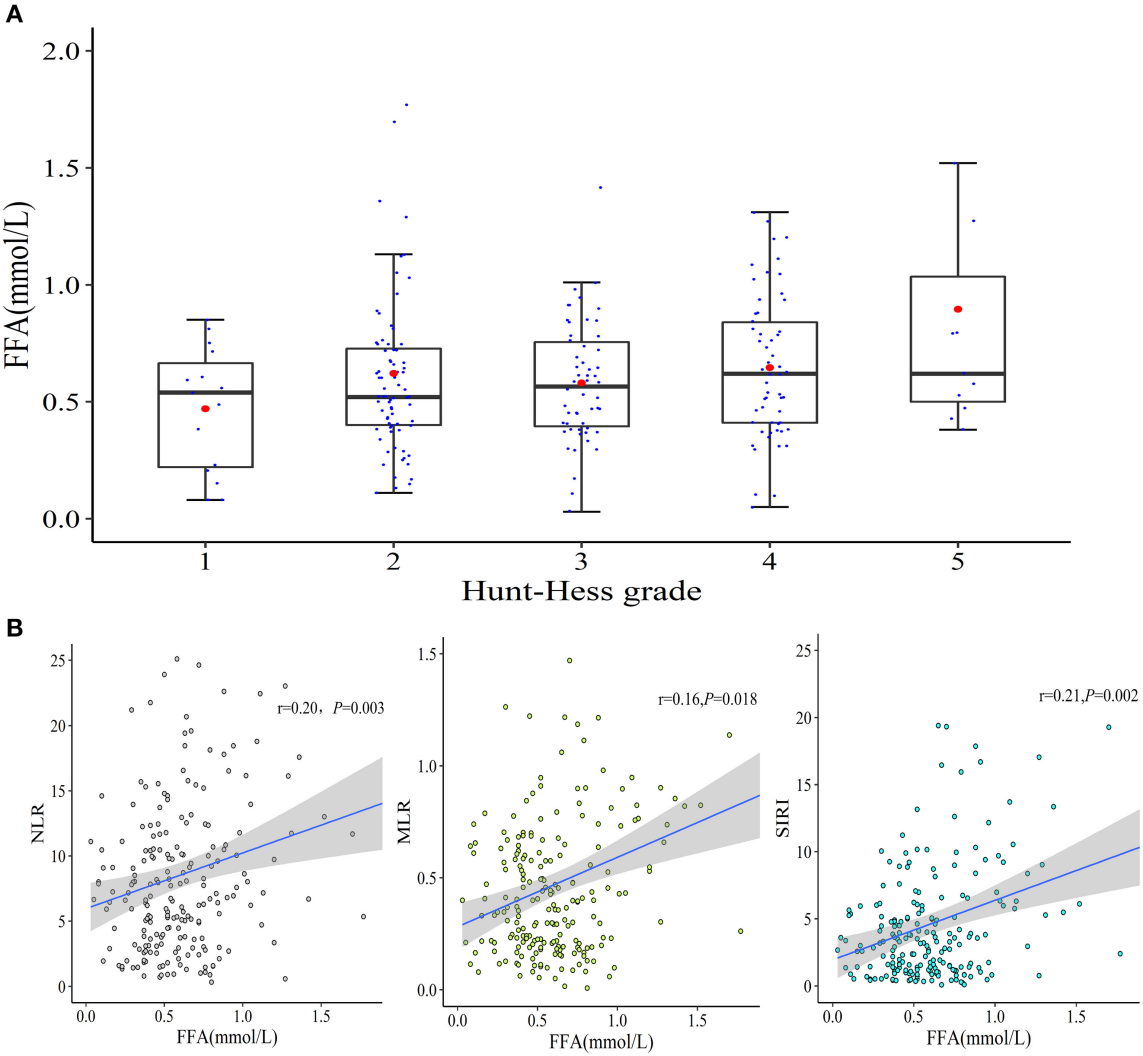
The correlations between admission FFA levels and aSAH characteristics. **(A)** A weak correlation between admission FFA levels and initial HH grade was observed (*r* = 0.141 and *P* = 0.04). **(B)** Correlations between serum FFA levels and NLR, MLR, and SIRI.

As shown in Table 1, the differences in TG, TC, HDL-c, and LDL-c levels between patients with good and poor outcomes were not significant and none of them had a significant association with the severity of aSAH. Meanwhile, the non-HDL-c level, non-HDL-c/HDL-c, or TG/HDL-c ratio had no prognostic effects on aSAH outcome following endovascular therapy. FFA levels on admission were higher in patients with poor mRS, but there was a low-grade correlation between the admission FFA and the initial severity of aSAH (Figure 2A) reflected by HH grade (*r* = 0.141 and *P* = 0.04). Collinearity was not found between age and serum lipid levels in the present dataset (Supplementary Table 1), as indicated by a tolerance >0.1 (=0.998) and VIF <10 (=1.002).

Based on the blood cell count data (Figure 2B), the increased FFA level on admission was positively correlated with NLR (*r* = 0.20 and *P* = 0.003) and MLR (*r* = 0.16 and *P* = 0.018). Furthermore, a prominent positive correlation between the FFA level and SIRI (*r* = 0.21 and *P* = 0.002) was detected.

### Predictors of Unfavorable Outcome

According to the univariable logistic analysis, patients of advanced age (*P* < 0.001), poor HH grade (*P* < 0.001 and OR = 5.8), high mFS (*P* < 0.001 and OR = 8.5), aneurysms located in the posterior circulation (*P* = 0.011 and OR = 3.5), and higher serum FFA levels on admission (*P* = 0.012 and OR = 2.5) were more likely to suffer poor outcome (Table 2). Subsequent multivariate logistic analysis results showed that age [adjusted OR (95% CI) = 1.0 (1.01.1), *P* = 0.034], HH grade [adjusted OR (95% CI) = 3.7 (1.87.7), *P* < 0.001], mFS [adjusted OR (95% CI) = 6.0 (2.912.5)*, P* < 0.001], aneurysms location [adjusted OR (95% CI) = 4.4 (1.315.0), *P* = 0.019], and FFA levels [adjusted OR (95% CI)= 3.1 (1.28.0), *P* = 0.021] were all verified as independent predictive factors for 3 months outcome of aSAH following endovascular therapy. In addition, the tolerance >1 and VIF <10 for the predictors suggested no collinearity among these independent variables (Supplementary Table 2).

**Table 2:** Univariate and multivariate logistic regression analyses of risk factors associated with 3 months poor outcome.

**Parameter**	**Univariate analysis**			**Multivariate analysis**		
	**OR**	**95% CI**	***P***	**Adjusted OR**	**95% CI**	***P***
Age	1.0	1.0–1.1	<0.001	1.0	1.0–1.1	**0.034**
Hypertension	2.1	1.2–3.6	0.009	0.9	0.4–2.0	0.857
HH grade (3–5)	5.8	3.2–10.7	<0.001	3.7	1.8–7.7	**<0.001**
mFS (3–4)	8.5	4.4–16.2	<0.001	6.0	2.9–12.5	**<0.001**
Posterior circulation	3.4	1.3–8.9	0.011	4.4	1.3–15.0	**0.019**
FFA	2.5	1.1–5.8	0.012	3.1	1.2–8.0	**0.021**

*HH, Hunt Hess; mFS, modified Fisher scale; FFA, free fatty acids; OR, Odds ratio; CI, confidence interval*.

*The bold value indicates that the P value of the parameter is less than 0.05*.

### Development of Nomogram and Predictive Values for the Poor 3-Months Outcome

**Figure 3:**
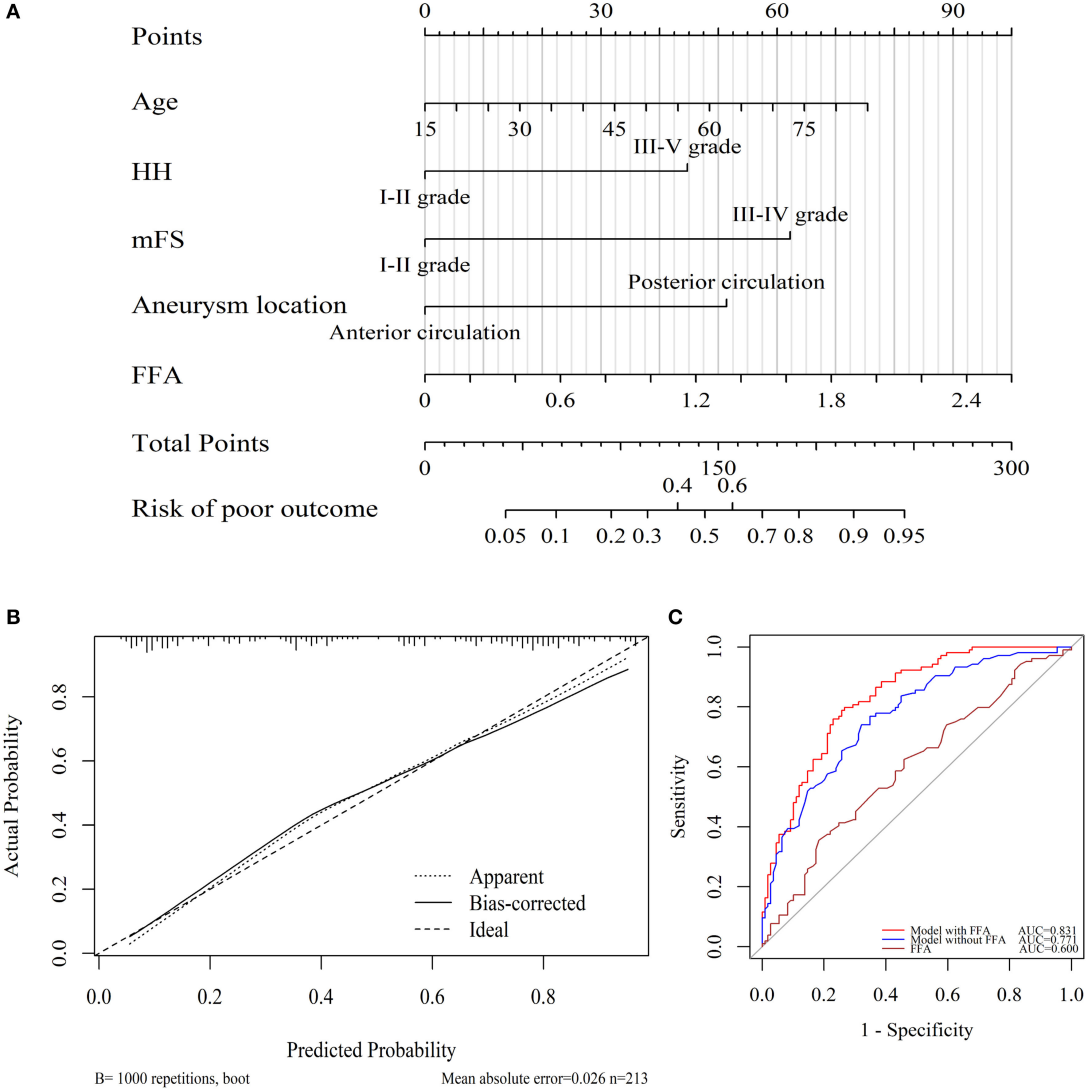
Utility of nomogram for prediction of poor outcome at 3 months after endovascular-treated aSAH. **(A)** Points were assigned for age, HH grade, modified Fisher grade, aneurysm locations, and serum FFA levels on admission. The “total points” are the sum of the individual score of five significant clinical variables. **(B)** The calibration curve of the nomogram for 3 months poor outcome. The red line of 45° represented an ideal prediction and the dotted line represented the performance of this nomogram (Hosmer-Lemeshow test *P* = 0.76). **(C)** ROC curves showed a better prediction of clinical factors incorporating FFA level than the model consisted of age, HH, mFS, and aneurysm location.

We further incorporated the independent predictors mentioned above into a nomogram (Figure 3A) to predict the probability of an unfavorable functional outcome. Each parameter was given an exact point. A higher total sum of the designated points in the nomogram refers to a higher risk of the poor 3-month outcome. The C-index of 0.831 (95% CI: 0.778–0.885) was calculated as the discriminative value of this nomogram, suggesting a good predictive power. In addition, the non-significant Hosmer-Lemeshow test result (*P* = 0.76) on the calibration curves indicated that the predicted outcomes matched well to the real unfavorable 3-month outcome observed (Figure 3B).

ROC curves regarding 3-month poor outcome were exhibited in Figure 3C. DeLong test verified that the FFA level alone had no predictive value (AUC = 0.600, 95% CI = 0.524–0.676, *P* = 0.012). The discriminatory accuracy for the model (HH, mFS, aneurysm parameters, and age) similar to the Changhai score (21) represented an AUC of 0.771 (95% CI: 0.708–0.833, *P* < 0.001), while the inclusion of FFA yielded a significantly enhanced AUC of 0.831 (95% CI = 0.778–0.885, *P* < 0.001), indicating a better capability of this model for the prediction of the poor aSAH outcome compared with the conventional aSAH outcome assessment.

### Validation of Clinical Application of the Nomogram

**Figure 4:**
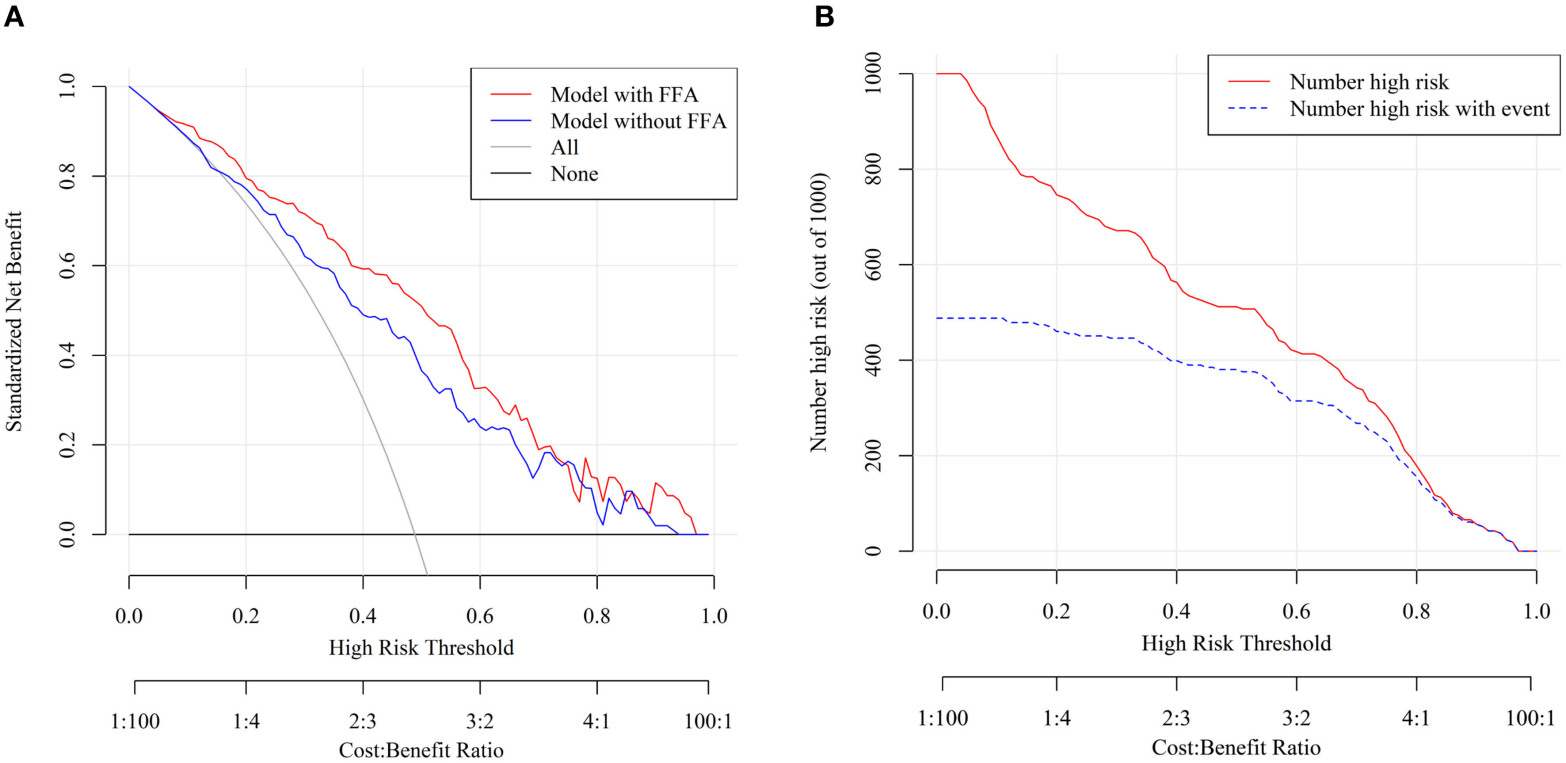
Decision curve analysis (DCA) and clinical impact curve of nomogram. **(A)** The x- and y-axes represented the threshold probability and the net benefit, respectively. The red line represented the nomogram. The blue line represented the assessment model constructed by age + HH + mFS + aneurysm location. The gray line represented the net benefit that all patients are treated. The black solid line represented the net benefit of the strategy of treating no patients. **(B)** The red curve showed the predicted number at different threshold probabilities and the blue curve represented the actual number of patients for a population size of 1,000.

Decision curve analysis was performed to investigate the net benefit of this predictive model (Figure 4A). The results suggested that the nomogram could provide greater net benefit than the models combining conventional components (age + HH + mFS + aneurysm location) when the threshold probability ranged from 5 to 75%.

Furthermore, the stratification of the unfavorable 3 months outcome probability for 1,000 patients was predicted using the clinical impact curve (Figure 4B). Of 1,000 patients, 580 were predicted to be at high risk, while 400 of 1,000 truly had poor functional outcomes when the tentative threshold was set at 40%. The cost-to-benefit ratio in this case was around 2:3.

**Figure 5:**
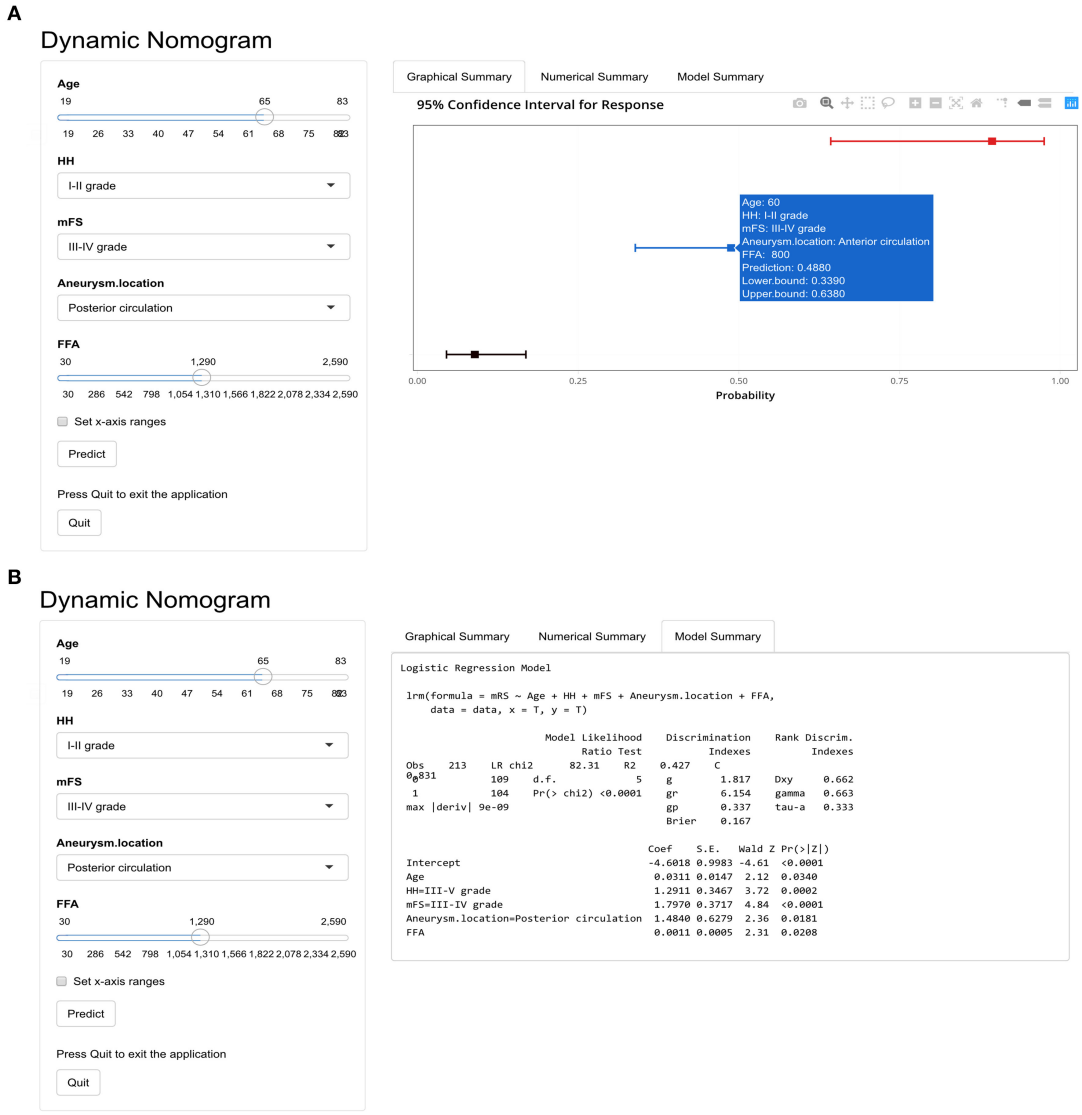
The online calculator for an individualized prediction of 3 months poor outcome based on the nomogram. **(A)** The output of graphical summary after calculation. **(B)** Model summary including the formula based on the logistic regression and parameters.

Based on nomogram, we created a dynamic online calculator (https://weiminwang-qdmh.shinyapps.io/DynNomapp/) to give perioperative predictions for the 3 months outcome. In this program, the unit of FFA is set at μmol/L to avoid decimal inputs. For example, if a 60-year-old patient with a HH grade of I–II and an mFS grade of II-IV was admitted to the emergency room due to the rupture of the anterior circulation aneurysm, the risk of developing a poor 3 months clinical outcome would be 48.8% (95% CI 33.9–63.8%) when the serum FFA concentration measured at admission was 0.8 mmol/L (800 μmol/L) (Figures 5A,B).

## Discussion

The key findings of the study include the following: (1) the advanced age, poor conventional scores (HH and mFS), and posterior circulation aneurysms (PCA) were strong predictors for unfavorable 3-month outcome in patients with aSAH receiving endovascular coiling; (2) the increased FFA level on admission was positively associated with the inflammation status and independently associated with the poor mRS after endovascular treatment; (3) the TC, TG, HDL-c, LDL, non-HDL-c concentration, and the ratios including non-HDL-c/LDL-c and TG/HDL-c had no significant association with the poor 3-month outcome. In addition, we established a novel nomogram model combining the admission FFA level to the conventional assessment components of aSAH outcome, which was demonstrated to have higher predictive accuracy than the conventional assessment tools.

Despite the consensus that the endovascular treatment for ruptured intracranial aneurysms is less invasive than open surgery, hemorrhagic (22), and ischemic (23) complications still occur, exerting deleterious effects on prognosis. The clinical outcome was affected by both the initial SAH severity and the preoperative conditions (24). Therefore, appropriate medical supports at the perioperative stage are critical to prevent the poor outcome. We retrospectively reviewed data from patients with aSAH and found that the age, HH grade, mFS, and aneurysm location were significant predictors for the 3-month outcome of a patient with aSAH receiving endovascular coiling. These characterizations were consistent with previous studies aiming to predict adverse outcomes following aSAH treatment (25, 26). For endovascular therapy, a grading system named Changhai score worked well on the aSAH clinical outcome prediction (21), of which the 4 pronounced risk factors including age, HH grade, mFS, and aneurysm location were identical to these findings. Although the HH grade and mFS have been widely used to evaluate the severity and outcome of aSAH in clinical practice, these evaluation scales have many limitations which could lead to the initial misdiagnosis due to the subjectivity of the neuro interventionalists or the adjudicated information (27, 28). Hence, it may be useful to take other preoperative factors into account for better predictions.

There has been increasing attention on FFA levels in multiple diseases. Biologically, FFA mainly originates from the hydrolysis of TG and serves as a preferred energy fuel for tissue (29). Acute critical illnesses, such as trauma or sepsis, could induce elevated FFA levels in serum (30). In cerebral diseases, FFA has been proven to be independently associated with both the poor prognosis and stroke recurrence in patients with ischemic stroke (31, 32); however, scarce data reveal the roles of serum FFA in aSAH outcome. This study found that the higher FFA serum level was an independent predictor for the poor 3-month outcome of aSAH following endovascular coiling, which is consistent with a previous study (33). Brain inflammation induced by FFA was reported as an important mechanism to compromise outcome in patients with SAH (34) and the activation of the FFA-triggered inflammation may result in severe neurotoxicity (35). This study found positive correlations between admission FFA levels in serum and inflammation biomarkers such as NLR, MLR, and SIRI, which indicated pro-inflammatory and immunosuppression status. Interestingly, we found no predictive values of TG, TC, HDL, and LDL on clinical outcome after aSAH treated by endovascular coiling in this study, although the HDL or LDL were widely reported to play multiple roles in aSAH.

As a visualization tool, nomogram has advantages over the conventional analysis using ORs (36). In the current study, we incorporated the admission serum FFA level with the independent risk factors to develop a nomogram for predicting 3-month outcomes in patients with aSAH after endovascular treatment. Compared with conventional methods, this nomogram combining FFA presented more accurate predictive efficacy, as the DCA and clinical impact curve illustrated well adoption of the nomogram into clinical use in terms of threshold probability (37). To the best of our knowledge, this is the first study to verify FFA as an indicator for brain inflammation and the poor outcome of the endovascular-treated patient with aSAH. Compared with NLR, PLR, or SIRI, the FFA levels are potent inflammatory indicators to present instant and direct information on prediction. Furthermore, to provide individualized estimation in clinical practice, we set up an online calculator to predict the 3-month outcome of such patients based on the nomogram. This calculator is also well-adapted to a mobile device with feasible browsing. The nomogram-assisted medical decisions may fulfill the demand for risk stratification of patients with aSAH and conducting risk-oriented therapy to improve outcome during the hospital stay.

This study has the following strengths: (1) the serum FFA level is an objective clinical parameter that can be easily measured; (2) the nomogram we established combining the admission FFA with the conventional risk factors showed improved prediction accuracy. However, several limitations are also found in this study: (1) this is a retrospective study based on single-centered analysis which might have a regional limitation; (2) although the internal validation showed that the discriminative value of nomogram was good, external validation from a different cohort is needed to further test the applicability of the nomogram; (3) certain confounders including the diet, drugs, or diseases may affect the FFA levels at pre-admission that were not completely under control.

## Conclusion

Admission FFA level in serum is an independent predictor of 3-month poor outcome for a patient with aSAH receiving endovascular therapy. Nomogram composed of age, HH grade, mFS, aneurysm location, and FFA levels serve good performance in predicting the risk of unfavorable 3-month outcome, which could guide the treatment strategies and subsequently improve the clinical outcome for patients with aSAH.

## Data Availability Statement

All data were open and available from the corresponding author on reasonable request.

## Ethics Statement

The studies involving human participants were reviewed and approved by the Ethical Committee of Qingdao Municipal Hospital. Written informed consent for participation was not required for this study in accordance with the national legislation and the institutional requirements.

## Author Contributions

YW and YX contributed to the analysis of data, preparing figures, and drafting the manuscript. TL and LL obtained funding. TL and WW were involved in study design, data analysis, and critical revision of the manuscript. HW, JZ, CL, FC, CR, ML, ZX, and LL were responsible for the data collection. All authors have read and approved the final manuscript.

## Conflict of Interest

The authors declare that the research was conducted in the absence of any commercial or financial relationships that could be construed as a potential conflict of interest.

## Publisher's Note

All claims expressed in this article are solely those of the authors and do not necessarily represent those of their affiliated organizations, or those of the publisher, the editors and the reviewers. Any product that may be evaluated in this article, or claim that may be made by its manufacturer, is not guaranteed or endorsed by the publisher.

## Abbreviations

aSAH: aneurysmal subarachnoid hemorrhage
AUC: areas under the curves
CI: confidence interval
C-index: concordance index
CT: computed tomography
DCA: decision curve analysis
DSA: digital subtraction angiography
FFA: free fatty acids
HDL-c: high-density lipoproteins cholesterol
HH: Hunt Hess
LDL-c: low-density lipoprotein cholesterol
MLR: monocyte-to-lymphocyte ratio
mFS: modified Fisher scale
NLR: neutrophil-to-lymphocyte ratio
non-HDL-c: non-high-density lipoprotein cholesterol
OR: Odds ratio
PCA: posterior circulation aneurysm
ROC: receiver operating characteristic
SIRI: systemic inflammation response index
TC: total cholesterol
TG: triglyceride
VIF: variance inflation factor.
