# Supplementary material for: Development and Clinical Translation of a Perioperative Nomogram Incorporating Free Fatty Acids to Predict Poor Outcome of Aneurysmal Subarachnoid Hemorrhage Following Endovascular Treatment
Source: Front Neurol. 2021 Jul 26;12:629997. doi: 10.3389/fneur.2021.629997 (PMC8354211; doi:10.3389/fneur.2021.629997)
Supplement: Supplementary file 2 [file Table_2.DOCX]

**Table 1. The tolerance and VIF for the age and serum lipid profiles.**

| Parameters | Collinearity statistics | |
| --- | --- | --- |
|  | Tolerance | VIF |
| Age | 0.968 | 1.033 |
| HDL-C | 0.911 | 1.097 |
| LDL-C | 0.940 | 1.063 |
| FFA | 0.979 | 1.022 |

**Tabel 2. The tolerance and VIF for the significant predictors.**

| Parameters | Collinearity statistics | |
| --- | --- | --- |
|  | Tolerance | VIF |
| Age | 0.872 | 1.147 |
| Hypertension | 0.794 | 1.260 |
| HH grade | 0.742 | 1.348 |
| mFS | 0.824 | 1.213 |
| Aneurysmal location | 0.983 | 1.018 |
| FFA | 0.971 | 1.030 |

**Tabel 3. Comparison of ROC curves using DeLong’s test**

| Model with FFA VS Model without FFA | |
| --- | --- |
| Difference between areas | 0.060 |
| Standard Error | 0.023 |
| 95% Confidence Interval | 0.013 to 0.108 |
| z statistic | 2.535 |
| Significance level | *P* = 0.011 |
